# Input–output organization of the mouse BLA-projecting IL neurons

**DOI:** 10.3389/fnins.2025.1532078

**Published:** 2025-02-19

**Authors:** Zhuo Li, Yu Qu, Qi-Lin Wu, Li Tang, Yuan Dong, Xu-Feng Xu

**Affiliations:** ^1^Basic School of Medicine, Qingdao Medical College, Qingdao University, Qingdao, China; ^2^Institute of Neuropsychiatric Diseases, The Affiliated Hospital of Qingdao University, Qingdao University, Qingdao, China; ^3^Qilu Hospital of Shandong University, Jinan, China; ^4^School of Rehabilitation Science and Engineering Qingdao National High-tech Industrial Development Zone, University of Health and Rehabilitation Sciences, Qingdao, China; ^5^Qingdao Institute of Measurement Technology, Qingdao, China

**Keywords:** the ventromedial prefrontal cortex, basolateral amygdala, AAV, rabies, neurons

## Abstract

**Introduction:**

The infralimbic cortex (IL), a critical subregion within the ventromedial prefrontal cortex (vmPFC), modulates emotion, memory, and social functions via robust projections to the basolateral amygdala (BLA). However, the afferent brain regions that innervate BLA-projecting IL neurons (IL-BLA PNs) and their efferent targets have not been systematically characterized.

**Methods:**

We employed tracing techniques integrating adeno-associated virus (AAV) and monosynaptic rabies to systematically investigate the presynaptic inputs and axonal outputs of IL-BLA PNs.

**Results:**

Our findings revealed widespread synaptic inputs to IL-BLA PNs from subcortical areas, with the strongest inputs originating from the dorsomedial thalamus (MD) and anterior medial thalamus (AM) in the thalamic region, as well as from the ventral hippocampus (vHPC) and piriform cortex (Pir) in the limbic system. Sparse labeling of the virus demonstrated that IL-BLA PNs collateralize to innervate various subcortical regions, including the horizontal diagonal band (HDB), lateral preoptic area (LPO), ventral pallidum (VP), and lateral hypothalamus (LH).

**Discussion:**

This work establishes essential theoretical frameworks for the functional investigation and targeted intervention in neurological disorders.

## Introduction

The infralimbic cortex (IL) is a key component of the ventromedial prefrontal cortex (vmPFC), serving as a central hub for regulating learning, memory, decision-making, and emotional processes such as anxiety and depression (Hiser and Koenigs, 2018; Jones and Graff-Radford, 2021). In numerous mammalian species, IL establishes connections with various cortical and subcortical regions to fulfill its regulatory functions (Porter and Sepulveda-Orengo, 2020). Consistent findings from cumulative studies have explored IL’s involvement in diverse functional regulations through distinct input and output circuits; for instance, IL exerts opposing effects on anxiety and fear by projecting to the lateral septum (LS) and central amygdala (CeA), respectively (Chen et al., 2021). Additionally, manipulation of the IL-NACsh projection influences decision-making behaviors in mice, while projections to the paraventricular thalamus (PVT) are implicated in fear memory extinction (Sweis et al., 2018). However, most studies have concentrated on one or two specific circuits of IL for functional validation; and comprehensive anatomical investigations into both input and output pathways—particularly those involving third-level networks—remain limited.

The basolateral amygdala (BLA) represents another critical brain region involved in emotion regulation, memory processing, and decision-making that exhibits robust reciprocal connectivity with IL (Huang et al., 2020; Vafaei et al., 2022). Studies indicate that IL-BLA PNs can modulate various functions, including anxiety disorders, depressive states, and fear memories—underscoring the significance of the IL-BLA circuit in emotional regulation (Huang et al., 2020). Nevertheless, systematic exploration of presynaptic inputs to BLA-projecting IL neurons (IL-BLA PNs) alongside their parallel efferent projections to other brain areas remains largely unexplored.

In this study, we aim to elucidate the brain regions providing afferent projections to IL-BLA PNs and identify efferent targets receiving parallel projections from these neurons. Our findings will enhance understanding of the afferent-efferent neural networks associated with IL-BLA PNs, thereby establishing a foundation for future functional investigations.

## Materials and methods

### Mice

CD1 male mice (2–3 months old) were purchased from the Vital River Laboratory Animal Technology Co., Ltd. (Beijing, China). All mice were kept as groups of 4 individuals per cage, and maintained on an ambient photoperiod (12-h light–dark cycle) at 26 ± 1°C. All mice had ad libitum access to standard rodent chow and clean water. All animal experiments were approved by the Animal Ethics and Experimentation Committee of the Qingdao University and conducted in accordance with NIH animal care and use guidelines.

### Virus

Virus for retrograde tracing: AAV-hSyn-SV40-NLS-Cre (serotype AAV2/Retro, titer: 5.97E+12 vg/ml) was purchased from BrainVT A (Wuhan) Co., Ltd. rAAV-EF1α-DIO-EGFP-T2A-TVA (serotype AAV2/9, titer: 2.5E+12 vg/ml), rAAV-EF1α-DIO-oRVG (19G) (serotype AAV2/9, titer: 2.33E+12 vg/ml) and RV-EnvA-ΔG-mCherry (titer: 1.0E+8 vg/ml) were purchased from Blinkies Biotechnology Ltd.

Virus for anterograde tracing: pAAV-hSyn-Cre-mCherry (serotype AAV2/Retro, titer: 5.5E+12 vg/ml) was purchased from BrainVT A (Wuhan) Co., Ltd. pAAV-TRE-DIO-FLPo (serotype AAV2/9, titer 9.72E+12 vg/ml) and pAAV-TRE-fDIO-IRES-tTA (serotype AAV2/9, titer 5.09E+12 vg/ml) were purchased from Shanghai Heyuan Biotechnology (Shanghai) Co., Ltd.

### Stereotactic surgery and virus delivery

CD1 mice were anesthetized by 4% isoflurane in air in an induction chamber and maintained on 1.5% isoflurane (in air) through a nose clip during surgery. Stereotactic surgeries were performed with a stereotaxic frame (David Kopf Instruments, Tujunga, CA). Briefly, an incision was made along the midline of the scalp to expose the skull. Sites of the injection were determined according to Allen Reference Atlas (Dong, 2007) and small holes were drilled on the skull. Virus was microinjected into the targeted areas using glass micropipette controlled by a microinjection pump (RWD, Life Science) at a rate of 30 nL/min. After injection, the micropipette was left in place for 10 min to prevent viral backflow.

For retrograde tracing of the afferent projections to the IL-BLA circuit, AAV2/Retro-hSyn-SV40-NLS-Cre (80 nL) was unilaterally injected to the BLA (ML: +3.4 mm, AP: −1.2 mm, DV: −5.0 mm). A total volume of 60 nL AAV helpers containing 1:1 mixture of rAAV2/9-EF1α-DIO-EGFP-T2A-TVA and rAAV2/9-EF1α-DIO-oRVG (19G) was ipsilaterally injected into the IL (ML: +0.3 mm, AP: +1.6 mm, DV: −2.6 mm). Three weeks after the injection of AAV helpers, an injection of rabies virus RV-EnvA-ΔG-mCherry (50 nL) was administered to the IL. Mice were sacrificed 1 week after the rabies virus injection.

For anterograde tracing of the collateral projections of IL-BLA PNs, AAV-hSyn-Cre-mCherry (80 nL) was unilaterally injected in the BLA of CD1 mice. A total volume of 60 nL AAV containing 1:9 mixture of pAAV-TRE-DIO-FLPo and pAAV-TRE-fDIO-IRES-tTA was ipsilaterally injected into the IL. Mice were sacrificed 3 weeks after virus injection.

### Immunofluorescence

Mice were anesthetized with 4% isoflurane in an induction chamber and perfused with 0.9% saline followed by 4% paraformaldehyde (PFA, w/v). The whole brain was harvested and fixed in 4% PFA overnight at 4°C. Fixed brains were transferred to 30% sucrose solution for cryoprotection. Coronal sections (40 μm) were cut throughout the entire brain. During the serial sectioning process, 4 sets of samples were collected in parallel by including 1 of every 4 slices into a sample set. For immunofluorescence staining, brain slices were blocked with 5% donkey serum in PBS containing 0.2% Triton X-100 (Sigma, V900502), incubated with an anti-cre primary antibody (Abcam, 1:1000 diluted in blocking solution) and subsequently incubated with an Alexa Fluor 488-conjugated donkey anti-rabbit IgG secondary antibody (Thermo Fisher, 1:500 diluted in PBS). Stained brain slices were mounted, and DAPI was used to visualize nuclear morphology.

### Cell and fiber distribution analysis

The images of one set of brain slices were scanned using a digital pathology section system (Olympus, Japan). To assess the number of cells providing monosynaptic input to IL-BLA PNs, every fourth coronal section (40 μm thick) was sampled throughout the entire brain and imaged at 10× magnification. The brain regions were identified based on the Allen Reference Atlas, and the mCherry^+^ cell bodies within each region were manually counted and expressed as a proportion of the total cell count for each individual animal. The normalized input profiles from *n* = 3 animals were subsequently compared.

To quantify the axonal output density of BLA-projecting IL neurons, every other third coronal section (40 μm thick) was collected across the entire brain and mounted in serial order. Brain regions containing fluorescently labeled axons and terminal boutons were identified and scanned at 10× magnification with consistent laser intensity. The grayscale difference between background and fluorescence was calculated using ImageJ. For each brain region, at least five sample areas were selected across multiple sections to generate an average pixel density corresponding to axonal fluorescence. The average values for each region were then expressed as a fraction of the total pixel density quantified for all regions in a given animal, generating a normalized projection density profile for *n* = 3 animals.

### Statistical analysis

GraphPad Prism 9 statistical software was used for statistical analysis. To systematically analyze the distribution of afferent-efferent projections of the IL region, percentages were first quantified from individual brain sections (at least three values per region) for each of *n* = 3 animals. Normality was assessed using the Shapiro–Wilk normality test, and all data were confirmed to have a normal distribution. Comparison of means was then performed by One-way ANOVA. Differences between datasets were considered significant if *p* < 0.05.

## Results

### BLA-projecting neurons in IL and potential afferent local neurocircuit within vmPFC

**Figure 1:**
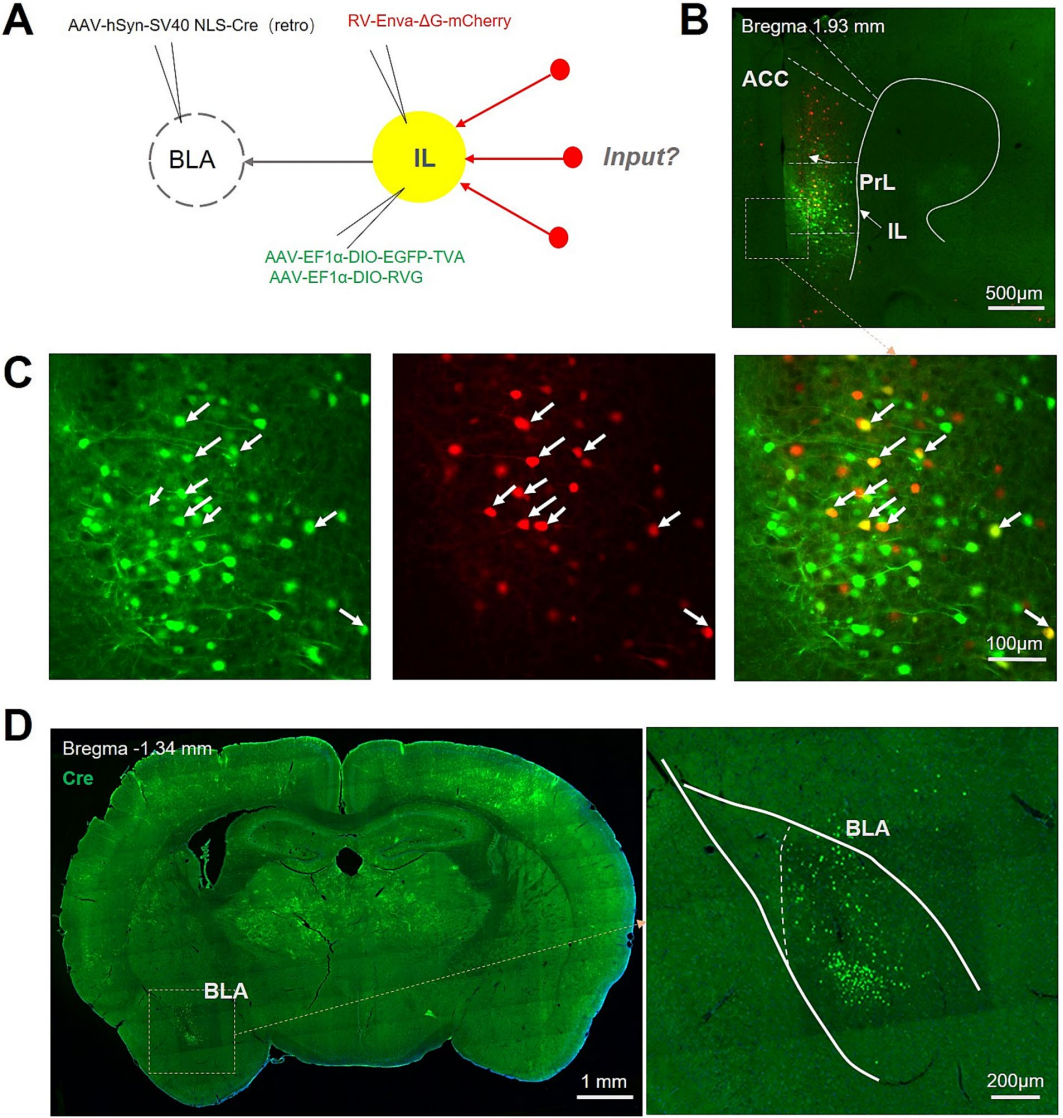
Experimental procedure for retrograde monosynaptic tracing of afferent projections to IL-BLA PNs. **(A)** Viral injection strategy for mapping pre-synaptic inputs to BLA-projecting IL neurons. **(B,C)** Example injection site showing the IL starter cell population (yellow) co-expressing rabies glycoprotein (RVG), TVA receptor, EGFP, and rabies-mCherry (red). Scale bar, Top 500 μm and bottom 100 μm. **(D)** Representative images show the Cre protein expression in BLA. Scale bar, left 1,000 μm and right 200 μm.

The mutual projection between BLA and IL has been previously reported. To further explore the afferent brain regions that participate in the regulation of this neurocircuit, we injected AAV/Retro-Cre into unilateral BLA and Cre-dependent helper viruses (AAV-DIO-EGFP-TVA and AAV-DIO-RVG, which enable retrograde transport of the rabies virus across the monosynapses) into the ipsilateral IL, followed 3 weeks later by an injection of rabies virus (RV-Enva-ΔG-mCherry) at the same site of IL, and another 1 weeks later by mice sacrifice and subsequent high-throughput whole-brain imaging (Figure 1A). We identified starter cells (neurons exhibiting co-expression of GFP and mCherry) confined to the IL (Figure 1B, white arrowheads) and Cre-positive neurons in the BLA (Figure 1C), suggesting the accurate localization of the IL-BLA PNs. Notably, a substantial number of mCherry-positive neurons localized in the prelimbic cortex (PrL) region, suggesting that IL-BLA PNs within the vmPFC receive input from the PrL (Figure 1B).

### Afferent brain regions of the IL-BLA PNs

**Figure 2:**
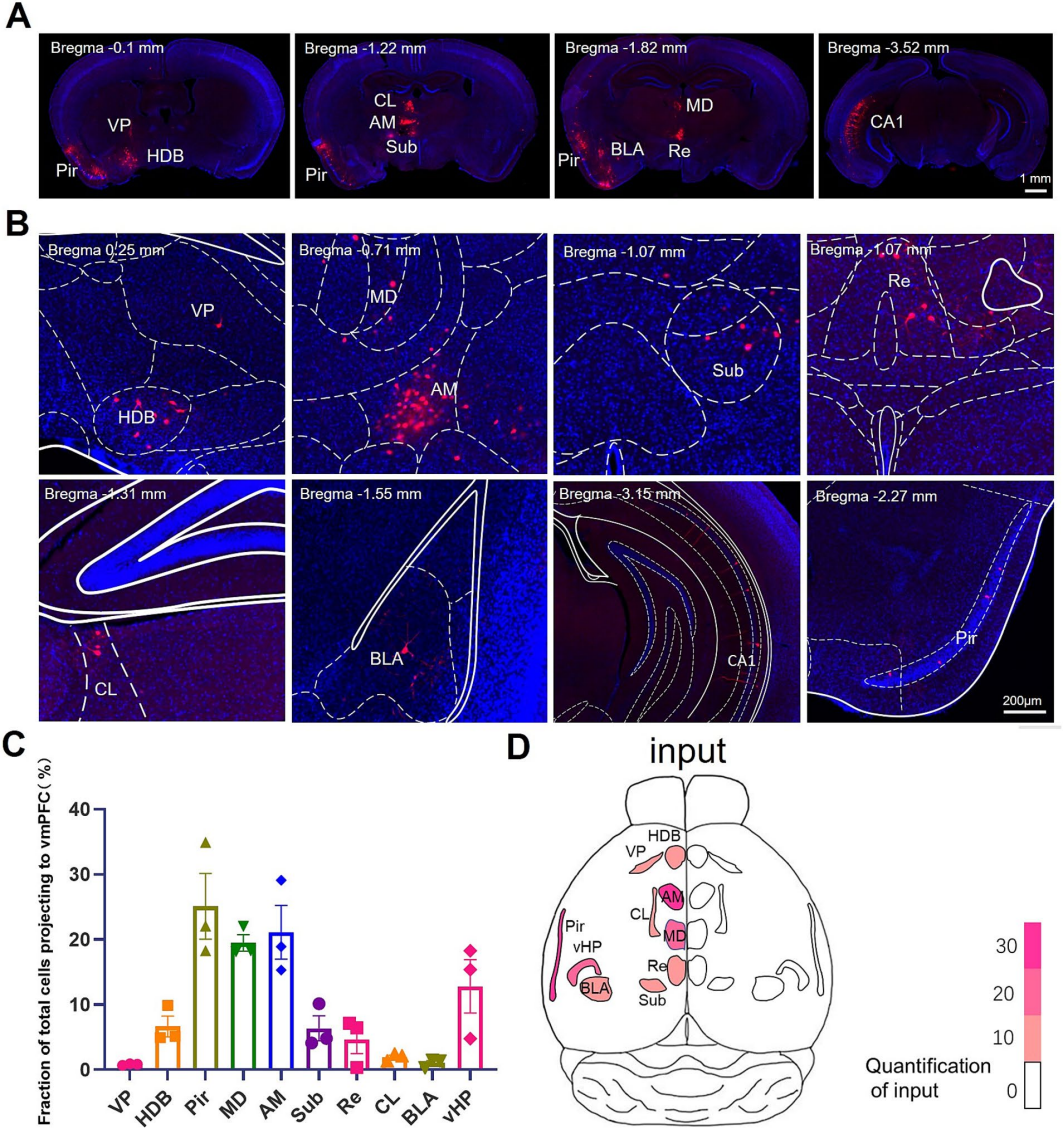
Monosynaptic inputs to BLA-projecting IL neurons **(A,B)**. Example images of presynaptic rabies-mCherry labeling (red) in HDB, VP, MD, AM, Sub, CL, BLA and CA1. **(C)** Quantification of presynaptic rabies-mCherry labeling in various brain regions. Data are presented as mean ± SEM. One-way ANOVA **(C)** [*F*_(9, 20)_ = 11.68, *p* < 0.0001]. **(D)** Overview of the whole brain distribution where pre-synaptic input to BLA-projecting IL neurons. AM, anteromedial thalamus; BLA, basolateral amygdala; CL, medial and lateral thalamus; HDB, diagonal horizontal limb; MD, dorsomedial thalamus; Pir, piriform cortex; Sub, inferior central thalamus; Re, thalamic nucleus combined canal; vHPC, ventral hippocampus; VP, ventral pallidum.

**Figure 3:**
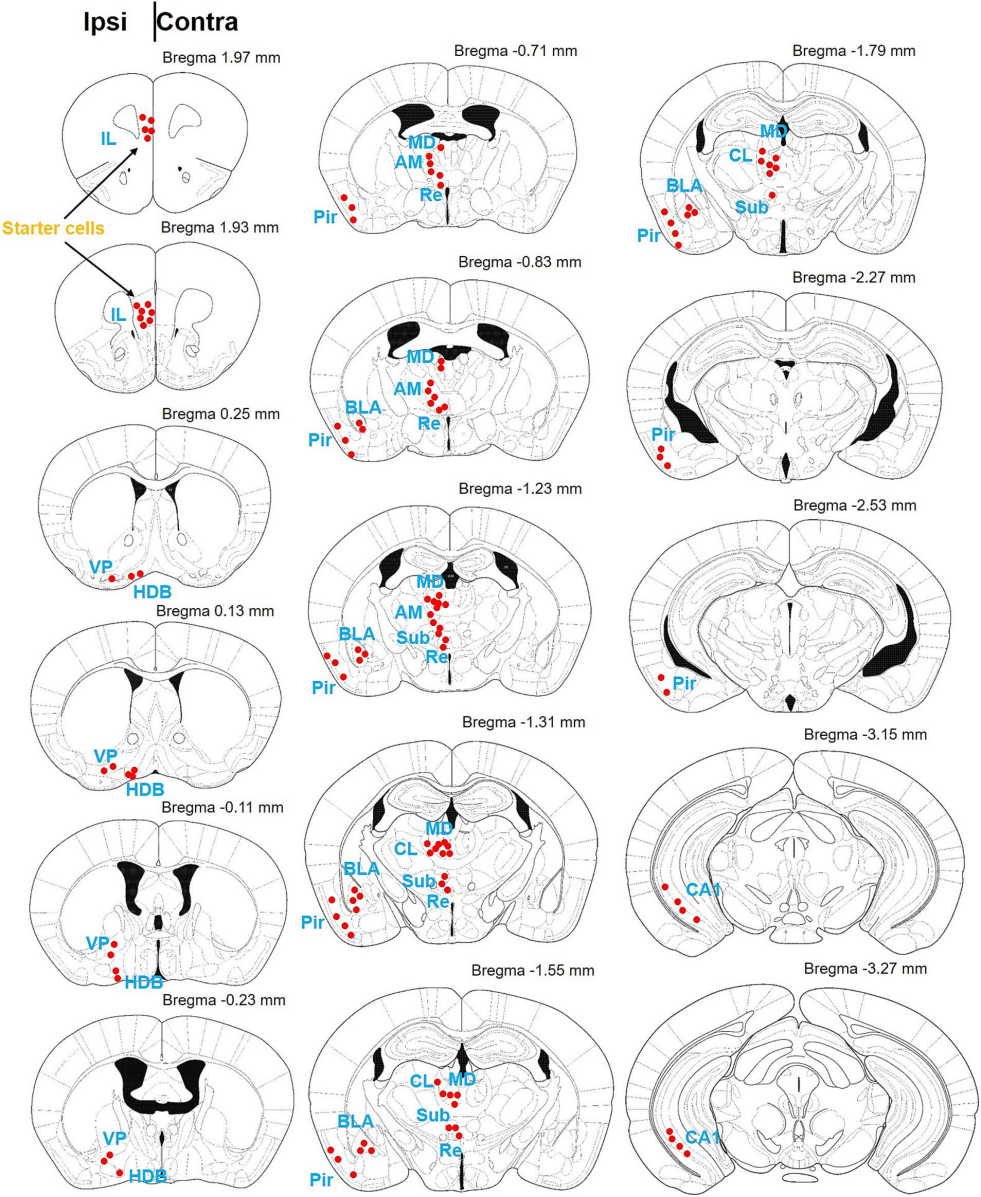
Schematic diagram of the brain map providing afferent projections to IL-BLA PNs. Summary of brain-wide inputs to BLA-projecting the IL neurons. Example labeling for one case plotted on corresponding coronal atlas sections. Red dots indicate location of retrogradely labeled cell bodies. AM, anteromedial thalamus; BLA, basolateral amygdala; CL, medial and lateral thalamus; HDB, diagonal horizontal limb; MD, dorsomedial thalamus; Pir, piriform cortex; Sub, inferior central thalamus; Re, thalamic nucleus combined canal; vHPC, ventral hippocampus; VP, ventral pallidum.

To investigate the afferent projections to the BLA-projecting IL neurons, we conducted the whole brain analysis of the mCherry^+^ neurons. We observed mCherry^+^ neurons in ventral pallidum (VP) and horizontal limb of the diagonal band of Broca (HDB) in the basal forebrain; the mediodorsal thalamic nucleus (MD) and anteromedial thalamic nucleus (AM) in the thalamus; the submedius thalamic nucleus (Sub), reuniens thalamic nucleus (Re), centrolateral thalamic nucleus (CL), BLA, ventral hippocampus (vHPC), and piriform cortex (Pir) (Figures 2A,B). The distribution of afferent projections to IL-BLA PNs was summarized in Figure 3. We then constructed a whole brain input list of IL according to the number of mCherry-labeled neurons normalized to total cell number of in certain brain region and found that IL-BLA PNs received most projection from the Pir, DM, AM and vHPC (Figures 2C,D). This finding demonstrated that the IL-BLA circuit received afferent projections from the above-mentioned brain regions.

Efferent brain regions receiving collateral projections from IL-BLA projection neurons.

**Figure 4:**
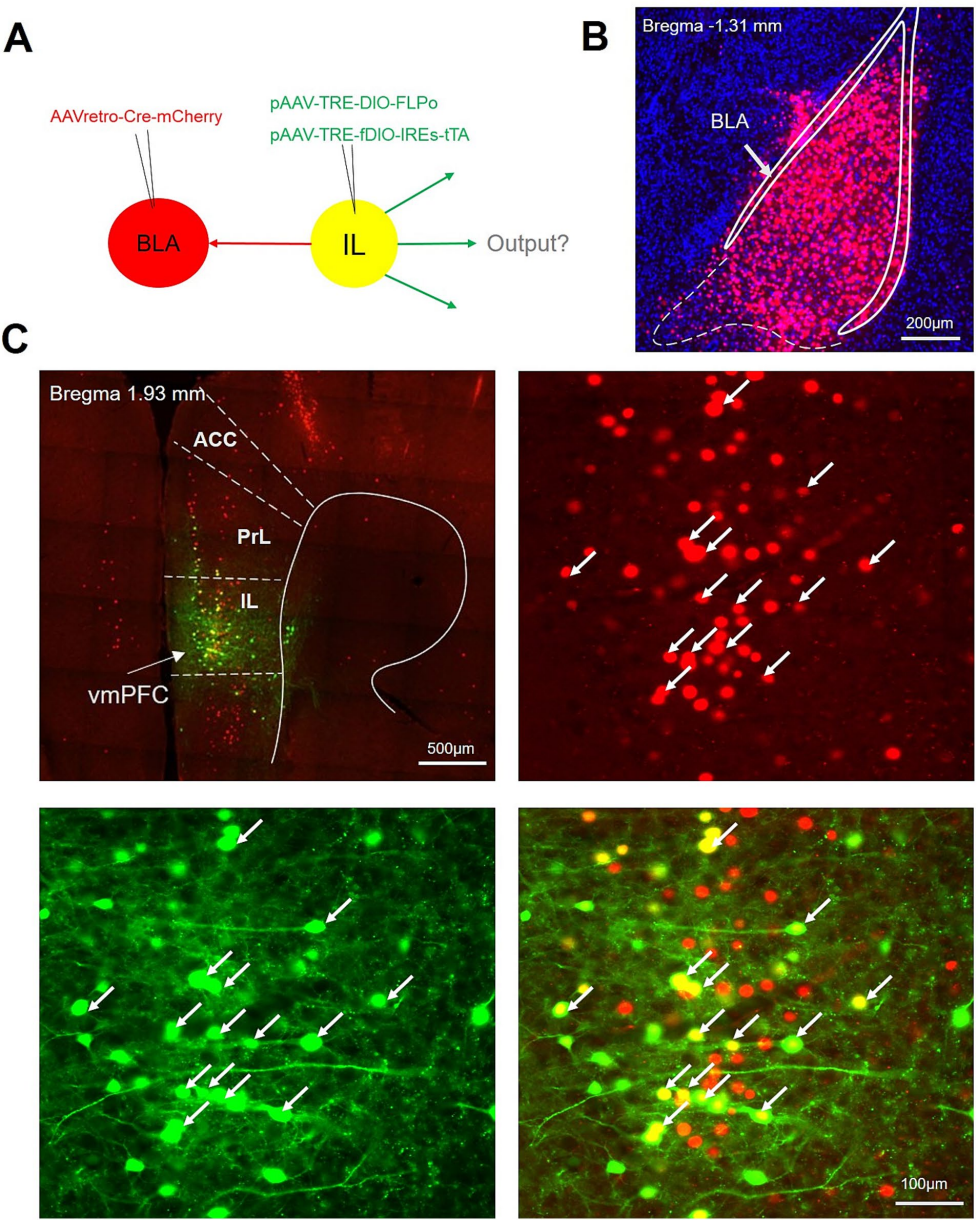
Experimental procedure to trace the collateral efferent output of IL-BLA PNs **(A)**. viral injection strategy for mapping collateral axonal output from BLA-projecting IL neurons. **(B,C)** Example injection site of AAV/Retro-Cre in BLA **(B)** and mixed sparse labeling virus in IL **(C)**. Scale bar, 200 μm **(B)**, 500 μm (**B** left top) and 100 μm (**C** right bottom).

**Figure 5:**
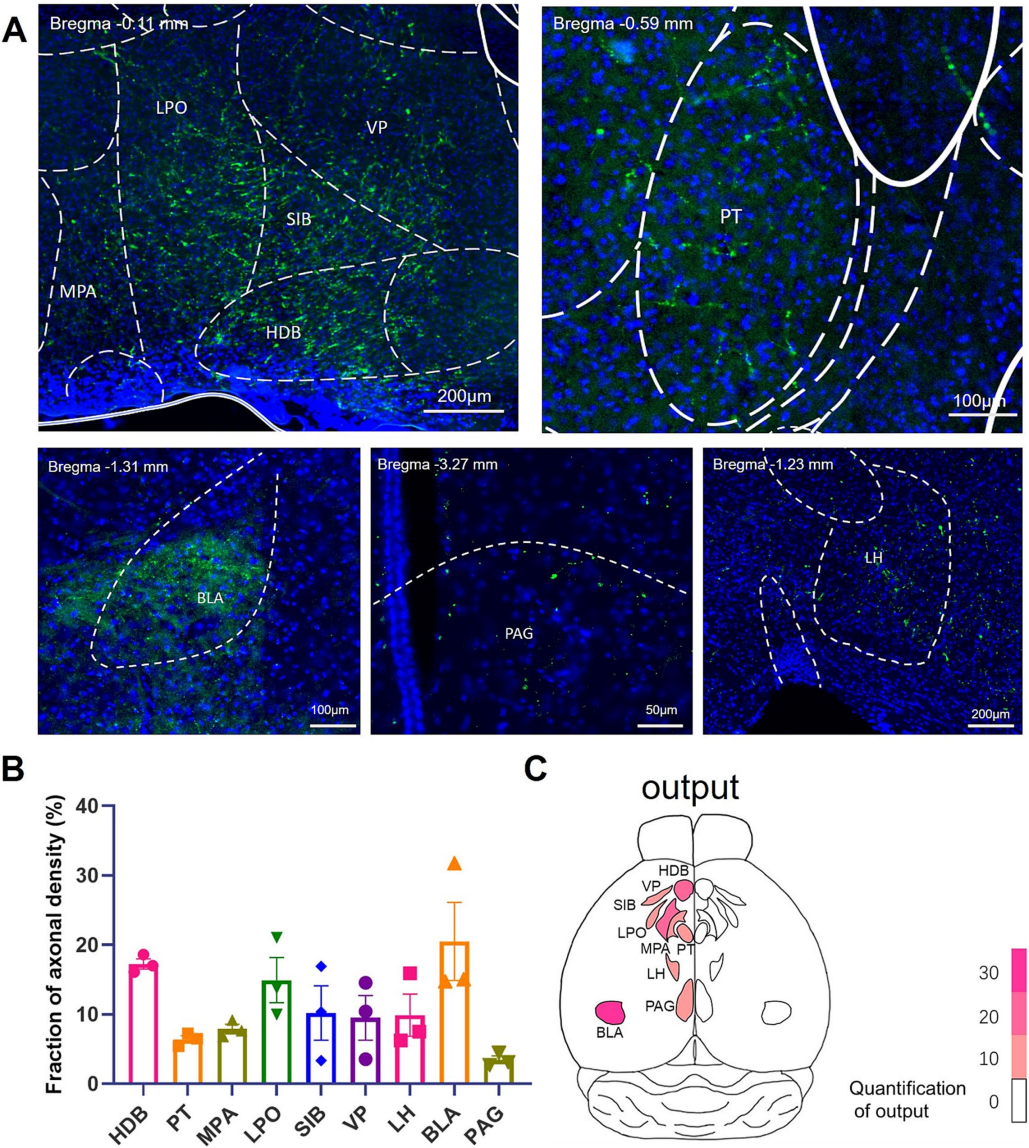
Brain regions receiving efferent projections from IL-BLA PNs example images of axonal projection labeling (green) in **(A)** MPA, LPO, VP, SIB, HDB, PT, PAG, LH, and BLA. **(B)** Quantification of postsynaptic EGFP labeling in various brain regions. Data are presented as mean ± SEM. One-way ANOVA **(C)** [*F*_(8, 18)_ = 4.046, *p* = 0.0066]. **(C)** Overview of the whole brain distribution where axonal output of BLA-projecting IL neurons. BLA, basolateral amygdala; HDB, diagonal horizontal limb; LH, lateral hypothalamus; LPO, lateral preoptic region; MPA, medial preoptic area; PAG, periaqueduct gray matter; PT, parabandal nucleus of the thalamus; SI, innomina; VP, ventral pallidum.

The IL has been reported to contain around 35% broadcasting neuron, which sent collateral projections to multiple brain regions (Morie et al., 2022; Hiser and Koenigs, 2018). We therefore intended to investigate the collateral projection pattern of the IL-BLA PNs (Figure 4A). Employing the recently developed sparse labeling approach, we injected the AAV/retro-Cre-mCherry into the unilateral BLA, which was retrogradely transported to IL cell bodies and turned on the Cre-dependent virus mixture of both controller (AAV-TRE-DIO-FLPo) and amplifier vectors (AAV-TRE-fDIO-GFP-IRES-Tta) injected into the ipsilateral IL (Figure 4A). We identified a confined population of mCherry-positive cells in the BLA and neurons co-expressing both EGFP and mCherry in the IL (Figures 4B,C), indicating that we have accurately delineated the IL-BLA PNs. For anterograde tracing of the collateral projections of the IL-BLA PNs, we analysed the axonal output density of BLA projecting IL neurons. GFP^+^ terminals were observed in the VP, HDB, substantia innominata (SI), medial preoptic area (MPA), lateral preoptic area (LPO), paratenial thalamic nucleus (PT), internal capsule (ic), the bed nucleus of the stria terminalis (BNST), BLA, the peduncular part of the lateral hypothalamus (LH), and he periaqueductal gray (PAG) (Figure 5A). Among these, the IL-BLA projection accounted for approximately 20% of the total IL collateral projection. Additionally, we found dense collateral projection from IL to HDB and LPO, accounting for 15 and 12% of the total projections, respectively (Figures 5B,C). This result suggested that the IL neurons may sent major collateral projection to BLA, HDB, and LPO.

### Significant co-localization of IL-BLA PNs and IL-LH PNs

**Figure 6:**
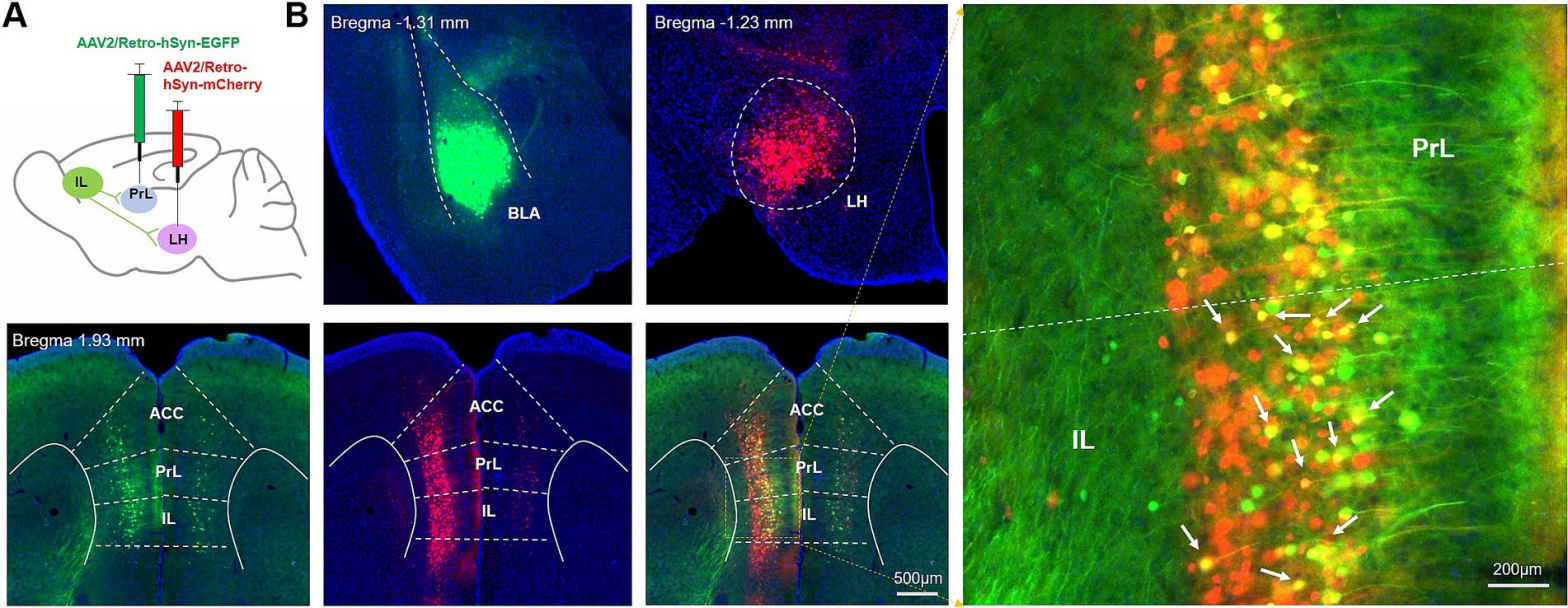
IL neurons projecting to the BLA are largely merged with those projecting to the LH. **(A)** Schematic sagittal sections show virus injections into the BLA and LH. **(B)** EGFP-expressing axons/terminals in the BLA and LH, mCherry-expressing axons/terminals in the LH, and IL locations of neurons labeled with retrogradely transported EGFP and mCherry. Scale bars, 200 μm.

Given the weak fluorescence intensity of neuronal terminals, which may introduce potential errors in our observations, we sought to further validate the accuracy of our pre-synaptic tracing results by employing retrograde AAV viral injections into downstream nuclei. Specifically, we administered a retrograde AAV virus expressing EGFP into the BLA and another retrograde AAV virus expressing mCherry into the LH—a brain region characterized by a substantial presence of GFP+ terminals in above data (Figure 6A). The findings revealed extensive co-localization between IL-BLA PNs and IL-LH PNs (Figure 6B), suggesting that numerous neurons within the IL are capable of projecting to both the BLA and LH, thereby confirming the reliability of our pre-synaptic tracing results.

## Discussion

**Figure 7:**
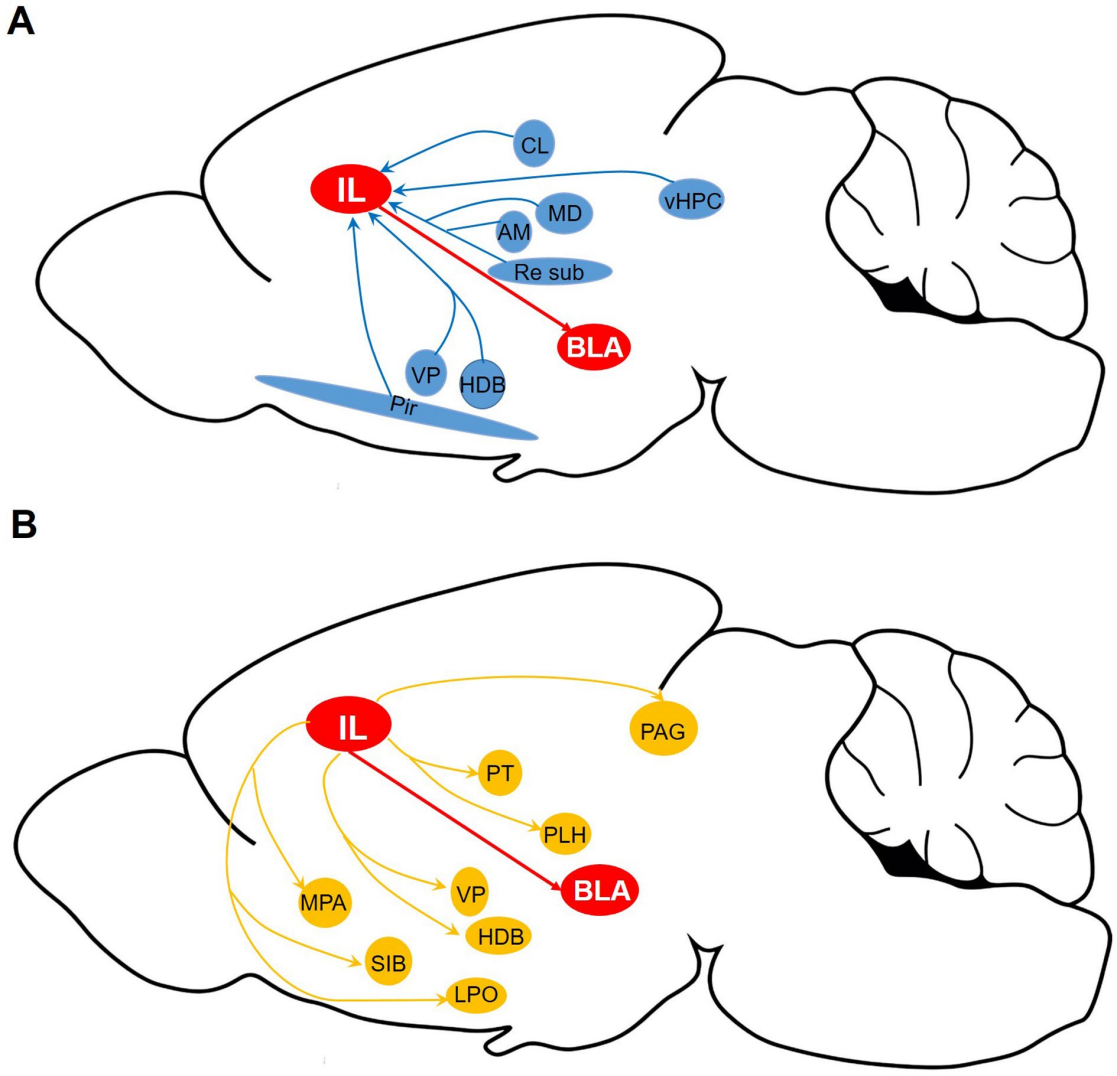
Whole-brain diagram of afferent and collateral efferent projections to and from IL-BLA PNs. **(A)** Monosynaptic input to BLA-projecting IL neurons. **(B)** Axonal output of BLA-projecting IL neurons. AM, anteromedial thalamus; BLA, basolateral amygdala; CL, medial and lateral thalamus; HDB, diagonal horizontal limb; LH, lateral hypothalamus; LPO, lateral preoptic region; MD, dorsomedial thalamus; MPA, medial preoptic area; PAG, periaqueduct gray matter; Pir, piriform cortex; PT, parabandal nucleus of the thalamus; SI, innomina; Sub, inferior central thalamus; Re, thalamic nucleus combined canal; vHPC, ventral hippocampus; VP, ventral pallidum.

The IL-BLA circuit is essential for the regulation of fear memory and emotional modulation; however, the synaptic input and output pathways of IL-BLA PNs remain inadequately explored. In this study, we conducted a comprehensive investigation of the pre-synaptic inputs and axonal outputs of IL-BLA PNs utilizing tracing techniques that combine adeno-associated virus with monosynaptic rabies. Our findings reveal extensive synaptic inputs to IL-BLA PNs from subcortical regions, including the AM, MD, Pir, vHPC (Figure 7A). The sparsely labeled viral tracing indicated that IL-BLA PNs exhibit collateral projections to innervate various subcortical areas such as the HDB, LPO, VP, and LH (Figure 7B). This work establishes essential theoretical frameworks for functional investigations and targeted interventions in neurological disorders.

IL is a pivotal subregion within the vmPFC, which regulates emotion, memory, and social functions through extensive projections to the downstream nuclei (Botterill et al., 2024). Several studies have systematically examined the input and output of the IL in rodents using retrograde or anterograde tracing techniques. These studies characterized that IL send widespread projections to the thalamus, hypothalamus, BLA, prelimbic and anterior cingulate cortices. Building upon this foundation, our study provides a comprehensive analysis of the input and parallel output circuits of IL neurons projecting to the BLA, further elucidating the structural network of IL neurons and contributing to a deeper understanding of their projection pathways. However, in our study, we did not extensively investigate the neuronal types involved in the projection to the IL-BLA PNs or the specific types of neurons that these IL-BLA PNs target. Future research should focus on a more comprehensive analysis of these neuronal populations to elucidate their roles in emotional regulation and circuit function.

Our results showed that IL-BLA PNs receive projections from the VP and HDB in the basal anterior brain region; the AM, MD, Sub, Re, Cl in the thalamus; the Pir and vHPC in the neocortical area. The VP is a central structure within the basal ganglia, serving as a key node in the brain’s reward circuit. It integrates reward signals primarily from the nucleus accumbens and regulates the execution of motivational behaviors, as well as modulates cognitive, emotional, and motor processes associated with motivational saliency. A large number of axonal tracing studies have shown that VP neurons project directly to the dorsal thalamus, lateral habenular nucleus, lateral hypothalamus, and ventral tegmental area and exert different roles through different neuronal types (Feng et al., 2021). HDB is involved in behaviors related to alertness, learning and memory, arousal, and attention through cholinergic neurons. Basal forebrain cholinergic neurons in HDB innervate the olfactory bulb (OB) and directly receive input from OB and multiple olfactory cortices. They are involved in olfactory information processing, arousal, attention, and learning (Zheng et al., 2022).

The thalamus is a central structure of the brain, composed of numerous neuronal populations that play vital roles in cognitive, emotional, sensory, and motor functions (Bordes et al., 2020). It is frequently implicated in the pathophysiology of neurological and psychiatric disorders. MD occupies a crucial position, enabling it to connect with various brain structures, particularly the prefrontal cortex, which is involved in various higher-order brain functions, such as learning and memory processes, and emotion (Morceau et al., 2022). The amygdala-dorsomedial thalamus-orbitofrontal cortex is involved in short-term memory and emotion (Gartner and Samuelsen, 2024). Changes in MD activity can affect emotion, such as increased cerebral blood flow observed in depressed patients (Gong et al., 2019). The ventral structure of AM can transmit information to the cortex-hippocampus-amygdala circuit, which is critical for fear memory processing (de Lima et al., 1991). Convergence pathways from the hippocampus, prefrontal cortex, and amygdala allow the thalamus to flexibly coordinate memory, cognitive, and affective activities disrupted in several psychiatric and neurological disorders in humans (Vertes et al., 2022). Pir is a significant component of the olfactory network located at the junction of the frontal and temporal lobes. It connects to many adjacent areas, and both animal and human studies have shown that the Pir is a key node in the temporal lobe epilepsy network (Chee et al., 2022). These brain regions have been extensively studied and verified to be involved in the regulation of emotions. Future research should focus on further elucidating the specific roles of these brain regions in emotion regulation.

We also utilized the Supernova system in the Luo lab to develop a sparse and bright labeling system for brain-wide specific neurons/neural circuits. Our brain-wide exploration revealed that the efferent brain regions receiving collateral projections from IL-BLA PNs include the VP, HDB, and SI, MPA, LPO, PT, ic, BNST, LH, and PAG. SI is associated with emotional processing, esp-ecially depression (Heimer et al., 1997). Recent studies have shown that SI transmits reward or aversion signals to the hypothalamus. The excitatory projection of LH by the SI is activated by the aversive stimulus to induce depressive-like behavior and inhibited by rewarding stimuli to inhibit depressive-like behavior (Cui et al., 2022). The preoptic region has long been considered the center of sleep and is also involved in regulating various innate functions, such as sexual behavior and parental behavior (Tsuneoka and Funato, 2021). Anxiety stressors elicit acute and prolonged responses in mouse MPA glutamate neurons, leading to the induction and expression of anxiety-like behaviors, especially in negative emotions such as anxiety and depression (Yamagata et al., 2021). Optogenetics activation of inhibitory neurons in mouse LPO triggers arousal, characterized by an activated electroencephalogram (EEG) pattern, suggesting increased arousal (Cao et al., 2024). Direct and indirect projections from the LPO to the ventral tegmental area (VTA) suggest that LPO can modulate VTA activity and reward-related behavior (Gordon-Fennell et al., 2020).In patients with depression, there is a pronounced disorder in the arrangement of local white matter in specific areas of the brain capsule, which is associated with psychomotor dysfunction (Hyett et al., 2018). Deep brain stimulation (DBS) targeting these regions can ameliorate major depressive disorder and severe obsessive-compulsive disorder (Widge et al., 2019).

The critical parts of the BNST, which extend the amygdala, are structurally and functionally connected to other limbic structures, including the amygdala complex, hypothalamic nucleus, hippocampus, and associated midbrain structures (Prakash et al., 2024). These connections play a crucial role in regulating various behavioral states, ranging from anxiety and reward processing to eating behavior (Ch'ng et al., 2018). Abnormal neuroplasticity and dysfunction of the BNST have been associated with chronic pain, depression, anxiety-related abnormalities, and other psychopathologies, including post-traumatic stress disorder (Hammack et al., 2021). PLH is essential for controlling eating behaviors and spontaneous physical activity (SPA) (Benfato et al., 2017). Damage or stimulation in this region can alter these behaviors (Morrison, 1968). Hypothalamic orexin neurons modulate feeding and SPA by affecting various systems and projecting to multiple brain regions (Mavanji et al., 2022). PAG regulates pain-related responses, autonomic function, analgesia, and stress-related behaviors. The role of PAG in the regulation of pain perception has been extensively studied, and it also a vital role in regulating chronic stress-induced depression-like behaviors (Zhang et al., 2024). This evidence suggests that these efferent brain regions receiving projections from IL-BLA PNs are closely associated with anxiety, depression, and pain-related negative emotions.

This study explored the afferent-efferent neural networks that regulate the IL-BLA emotional regulation circuit from the perspective of viral tracing and neural circuits. Extensive literature indicates that these afferent-efferent brain regions are involved in emotional regulation; however, the precise mechanisms by which they regulate emotion and the molecular pathways underlying these processes remain unclear. With advancements in optogenetics, chemogenetics, and electrophysiology, increasing evidence is emerging to elucidate the role of these brain regions in emotional regulation from both behavioral and electrophysiological perspectives. Rigorous clinical studies are also needed to provide a higher level of evidence for the neurological roles of the mPFC, vHPC, and PAG in regulating these brain regions during emotional processing.

## Data Availability

The original contributions presented in the study are included in the article/supplementary material, further inquiries can be directed to the corresponding authors.
